# The Mechanical Properties and Chloride Resistance of Concrete Reinforced with Hybrid Polypropylene and Basalt Fibres

**DOI:** 10.3390/ma12152371

**Published:** 2019-07-25

**Authors:** Xinyu Hu, Yihong Guo, Jianfu Lv, Jize Mao

**Affiliations:** College of Aerospace and Civil Engineering, Harbin Engineering University, Harbin 150001, China

**Keywords:** polypropylene fibre, basalt fibre, mechanical properties, synergy effect, chloride resistance, interface, pore structure

## Abstract

This paper aims to investigate the effect of the polypropylene fibre (PP) and basalt fibre (BF), singly or in hybridization, on the workability, mechanical, chloride resistance and pore structure characteristics of concrete. Sixteen mixtures consisting of PP and BF, both at volume content of 0.0, 0.1, 0.2 and 0.3%, were fabricated, and the slump, compressive, splitting tensile, flexural and charge passed were tested. The results show the hybridization of the PP and BF can improve three types of strength of concrete in comparison to their single fibre. Nevertheless, the hybridization is not always conducive, and the synergy of fibres is proposed and divided into positive and negative effects. The combination of the PP and BF both at content of 0.1% achieves the best mechanical performance, and is recommended for practical usage. Incorporating fibres reduces the chloride resistance of concrete, and the hybridization is helpless to this phenomenon; even the reduction is intensified at a highly hybrid fibre volume. However, increasing the curing age can mitigate this adverse effect caused by fibres. Furthermore, the microstructures were explored to elucidate the macro-properties of concrete in terms of interface and pore structure.

## 1. Introduction

Concrete is the most commonly used material in the civil engineering construction due to its high compressive strength, easy production and low-cost characteristics. Simultaneously, concrete is also considered a brittle material, which is limited in poor tensile strength and low ductility [1,2,3]. Effective measures that mitigate this limitation usually involve the addition of fibres to the concrete [4,5]. In the early 1960s, fibre-reinforced concrete (FRC) was brought to the attention of academic and industry research scientists [6]. Nowadays, FRC constructions have become increasingly widespread around the world, and there is still a high level of interest in the development of FRC [7,8,9]. Randomly dispersed fibres can effectively arrest the formation and propagation of cracks due to the bridge-action. However, the addition of a single fibre can be effective only in a specific range of crack dimension, depending on the fibre type, diameter, length and elastic modulus [10]. To better exert the bridge-action of fibre across the cracks, the idea of the hybridization of two or more fibres with different types and sizes is developed.

Among the various types of fibres, the hybridization of steel fibre and polypropylene fibre (PP) has been studied by many researchers [11,12,13,14,15,16,17,18,19,20]. Shan et al. [14] showed that the hybridization of steel fibre and PP resulted in superior mechanical performance of concrete compared to their individual fibre. Farhad et al. [15] indicated that the compressive strength and elasticity modulus of the hybrid fibre self-compacting concrete at the age of 91 days were higher than those of the single steel fibre and PP self-compacting concrete. Vahid and Togay [16] reported that the incorporation of steel fibre and PP improved the mechanical properties of high-strength concrete, and the best performance was achieved in the mixture which containing 0.85% steel fibre and 0.15% PP. Moreover, the hybridisation of steel and PP can improve the load bearing capacity [18], impact resistance [19,20], electrical resistivity [16] of concrete in comparison to concrete fabricated with single steel fibres. Nevertheless, steel fibre is very susceptible to corrosion, and increases the weight of structure due to high volumetric density; these disadvantages limit its usage [21].

Basalt fibre (BF), a kind of inorganic fibre obtained from basalt rocks, exhibits good physical and chemical properties. BF has higher tensile strength than PP, better chemical durability and thermostability than steel fibre [22]. These features, combined with the low cost and environmentally friendly characteristics, make BF a good alternative reinforcement in concrete composite materials [23]. Many studies have been performed on the compressive, splitting tensile, flexural, dynamic and fatigue performance of concrete reinforced with basalt fibre; and indicated a higher tensile strength, higher flexural strength, and better toughness and impact performance than those of plain concrete [23,24,25,26,27]. Using BF instead of steel fibre, and in combination of PP forming polypropylene-basalt fibre-reinforced concrete (PBFRC) has potential possibilities and attracts great interests. PP has a higher elongation at break, more than six times greater than that of BF, but the elastic modulus of BF can reach 88.9 GPa, which is far larger than 3.72 GPa of PP as shown in Table 2. BF and PP can form complementary advantages in the concrete matrix, and compensate for the defects of each single fibre, which results in substantially enhancing the tensile strength of concrete [28].

However, the research on the properties of PBFRC is limited [9,28,29]. Fu et al. [29] showed that the combination of PP and BF increased the energy absorbing property of concrete in comparison to each individual fibre, and the greatest energy absorbing property was twice than that of plain concrete. Smarzewski [9] investigated the influence of hybrid basalt and polypropylene fibres on the performance of high-performance concrete (HPC); and showed that the addition of the two types of fibres caused a reduction of the compressive strength, had negligible influence on flexural strength, but significantly enhanced the tensile strength and fracture energy. Wang et al. [28] indicated that the hybridisation of PP and BF considerably improved the flexural strength and splitting tensile strength of HPC compared to their single fibre. The mixture containing 0.15% BF and 0.033 PP achieved the best performance, and compared with HPC with no fibres, the compressive, flexural and splitting tensile strength increased by 14.1%, 22.8% and 48.6%, respectively.

Obviously, the published papers are far from sufficient and lack any in-depth analysis of the synergistic effect of PP and BF. Besides the mechanical performance, the permeability is also considered to be the most important feature for the long-term performance of concrete and needs accurate determination [30,31]. However, to the authors’ knowledge, no research has been performed on the permeability of PBFRC. Even systematic study on the chloride resistance of the single polypropylene or basalt fibre concrete is scarce and no consistent conclusions have been reached. Vahid et al. [32] and Algin and Ozen [33] indicated the addition of PP or BF both increased the resistance of concrete to chloride penetration. Conversely, Toutanji et al. [34] and Sadrmomtazi et al. [35] showed that concrete fabricated with PP or BF had lower chloride resistance than plain concrete.

From the above, studying the properties of PBFRC is of significant importance to extend the knowledge of the increasing use of FRC, and help engineers design and utilize it practically. The objective of this paper is to study the workability, compressive, splitting tensile, flexural, chloride resistance, interface and pore structure of concrete reinforced with polypropylene and basalt fibres. Sixteen mixtures consisting of PP and BF both at volume content of 0.0, 0.1, 0.2 and 0.3% were fabricated and cured for 28 and 56 days. Slump, compressive, splitting tensile, flexural, rapid chloride permeability (RCP), scanning electron microscopy (SEM) and mercury intrusion porosimeter (MIP) tests were applied and analysed in detail. Finally, the optimum PP and BF volume content was obtained and provided as a reference for design.

## 2. Experimental Program

### 2.1. Materials

**Figure 1:**
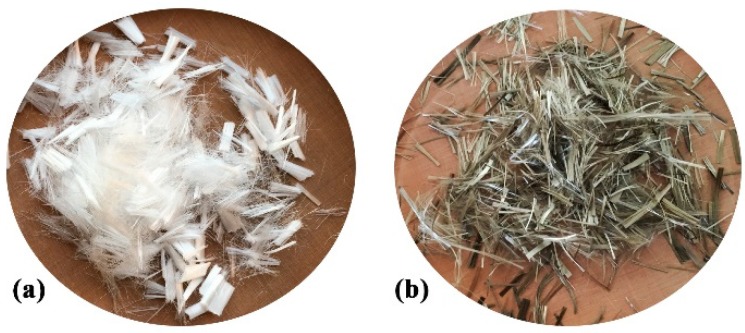
Fibres used: (**a**) PP; (**b**) BF.

Ordinary Portland cement (P·O 42.5) produced by the YaTai company of Harbin in China was used in this paper. Continuously graded gravel with size of 5–20 mm was used as coarse aggregate; river sand with fineness modulus of 2.7 was used as fine aggregate. A polycarboxylate super-plasticizer (SP) was also applied to meet the need for fluidity. Table 1 summarizes the chemical composition and main physical properties of cement. The PP and BF used are shown in Figure 1, and the physical and mechanical properties provided by the manufacturer are listed in Table 2.

### 2.2. Mix Proportions

The details of the sixteen mixtures are presented in Table 3. The water–cement ratio was maintained as 0.32 for all mixtures, and all the concrete components were the same, excluding fibres. The PP and BF both were at volume content of 0.0, 0.1, 0.2 and 0.3%, and the abbreviation for mixtures was named based on the content. For instance, PP01BF02 means that the volume content of PP is 0.1% and the volume content of BF is 0.2%.

### 2.3. Test Procedure

**Figure 2:**
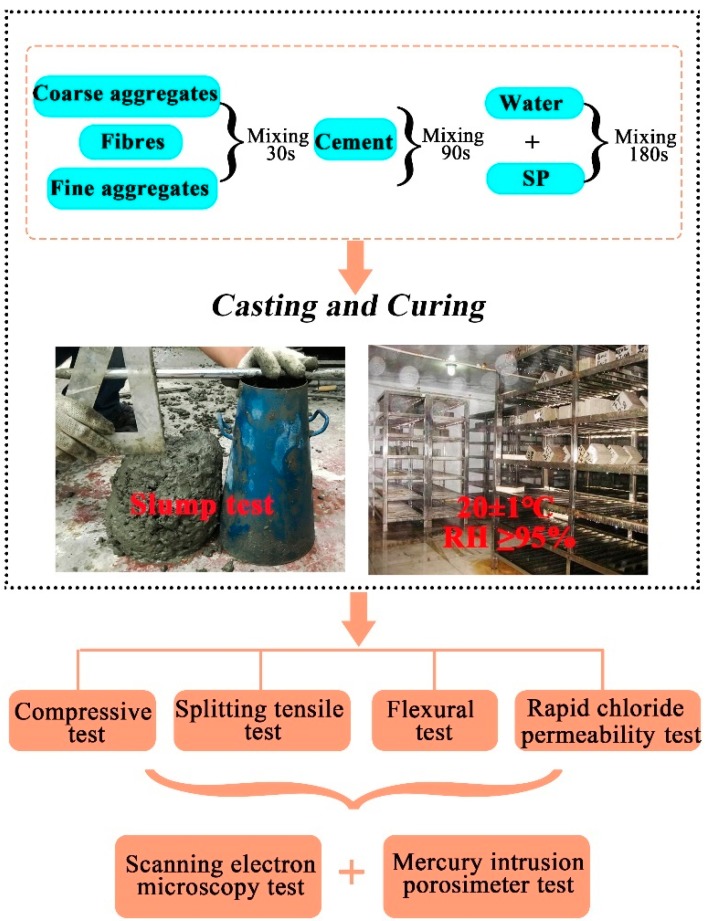
Diagram of the test program.

A diagram of the test program is shown in Figure 2. From each mix, three cubic specimens 100 mm × 100 mm × 100 mm, three cylindrical specimens Ø150 mm × 300 mm, three prismatic specimens of b = 100 mm width, h = 100 mm height, and L = 400 mm length and three cylindrical specimens Ø100 mm × 50 mm were used for compressive, splitting tensile, flexural and RCP testing, respectively. To eliminate the difference between the mixtures due to uneven dispersion of fibres, the mixing procedures strictly followed the first step shown in Figure 2. After casting, the moulds were filled with fresh concrete and vibrated on a shaking table until no air bubbles were visible, but for no more than 90 s. Then, all mixtures were covered with a plastic sheet to prevent them from dripping water out. Samples were demoulded after 24 h, and cured for 28 and 56 days at standard curing room (20 ± 1 °C and ≥ 95% RH).

After curing, the specimens were removed from the curing room and the mechanical properties were tested in a time period of approx. 1 h. The YAD-3000 test apparatus produced by ChangChun Kexin company (ChangChun, China) was applied for compressive and splitting tensile test, the Instron 5500R electro-hydraulic universal test machine was used for flexural test. All the mechanical tests were executed according to Chinese code GB/T 50081 [36], and the loading rate of the machine was 0.8 MPa/s.

**Figure 3:**
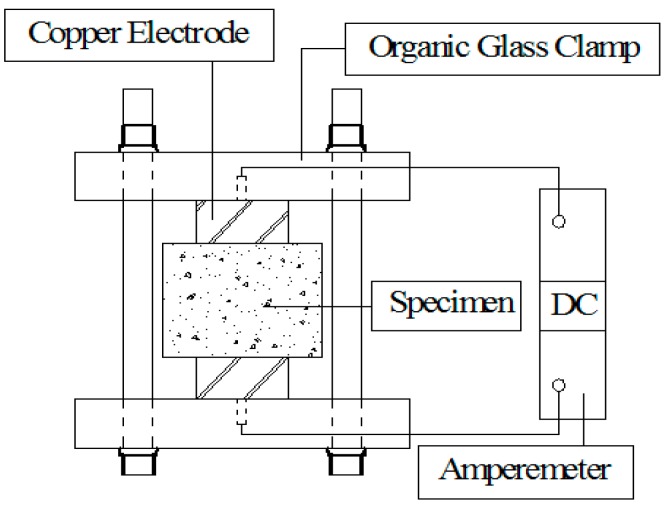
Schematic diagram of the rapid chloride permeability (RCP) test.

In order to evaluate the chloride resistance of concrete, the rapid chloride permeability (RCP) test was adopted and conducted in accordance with the procedures described in ASTM C1202 [37]. After 28 or 56 days, the specimens were taken from the curing room and put in a vacuum saturation apparatus for saturation. Thereafter, the specimens were placed into the testing cells: one of the cells connected to the power-supply cathode was full of 3% NaCl solution, and the other cell connected to the power-supply anode was filled with 0.3 mol/L NaOH solution. A direct voltage of 60 ± 0.1 V was continuously applied during the tests, and the total charge passed was recorded and used as an indicator of the chloride resistance of concrete. A schematic diagram of the experimental setup is shown in Figure 3.

Scanning electron microscopy (SEM) was performed to analyse the morphology of fibres in the concrete matrix by using Quanta 200 produced by FEI company (Eindhoven, Netherlands). The samples were taken from broken specimens after mechanical tests and crushed into small pieces, followed by drying in a vacuum desiccator at 50 °C until a constant weight was reached. Finally, a thin gold layer was coated on the concrete pieces before testing was conducted.

The mercury intrusion porosimeter (MIP) test was adopted to analyse the pore structure of concrete, using the AutoPore IV 9500 test machine manufactured by Micromeritics company (Atlanta, GA, America). Significant characteristic parameters, such as the pore diameter, porosity and mode pore diameter, can be concluded from MIP results. The samples were taken randomly from the specimens and broken into pieces of approximately 3–5 mm, and the weight of the pieces was approximately 4.5 g. Before testing, the samples were placed into a vacuum desiccator and dried at 60 ± 1°C for 24 h. Excessive drying temperature will damage the original pore structure of concrete.

## 3. Result and Discussion

### 3.1. Slump of Fresh Concrete

The slump results for all mixtures are presented in Table 3. The addition of fibres, singly or in hybridization, reduces the slump value of concrete to a certain extent. The slump values of mixtures are within the range of 50–130 mm. The control mixture has the highest slump value. In addition, the hybridization of PP and BF has no significant influence on the workability of concrete compared to their single fibre at the same content. The hybrid mixture PP03BF03 which containing 0.3% PP and 0.3% BF reduces the slump value by 61.54% compared to the control mixture.

The same slump reduction in concrete due to fibre addition has been reported by other researchers [23,38,39]. The reasons can be summarized as follows: first, fibre has a high surface area and needs more cement paste to cover its surfaces [40], which leads to the increase of the viscosity of concrete; second, the network structure formed by fibres increases the internal binding force of the matrix and restrains the mixture from segregation and flow. Moreover, the hybridization of fibres at a high volume increases the possibility of entanglement, which results in a more obvious slump reduction.

The incorporation of PP in concrete at volume fractions of 0.1, 0.2 and 0.3% reduces the slump value by 23.08, 34.62 and 50%, respectively. The presence of BF at volume fraction of 0.1, 0.2 and 0.3% causes slump reduction by 7.69, 19.23, and 38.46%, respectively. This indicates a more tangible slump reduction in concrete reinforced with PP compared to BF at the same volume fraction. This can be attributed to the higher surface and a larger size of PP than BF.

### 3.2. Compressive Strength.

**Figure 4:**
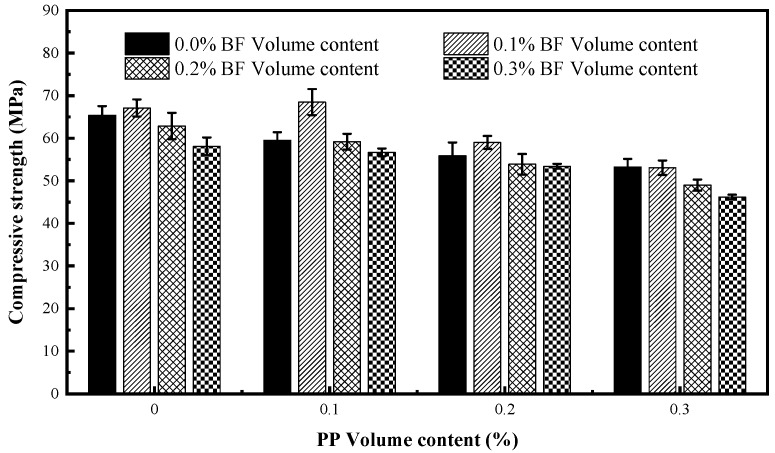
Compressive strength of fibre-reinforced concrete.

The compressive strength of mixtures including different fibre volumes is shown in Figure 4.

The results show that incorporating PP alone decreases the compressive strength of concrete and the addition of the BF alone at volume content of 0.1% slightly increases the compressive strength. Compared with the control mixture, the compressive strength of concrete reinforced with PP at volume fractions of 0.1, 0.2 and 0.3% reduces by 9.02, 14.54, and 18.56%, respectively. Banthia and Soleimani [41] reported a 13% reduction in the compressive strength of concrete reinforced with PP in comparison to no-fibre concrete. Sun [42] indicated that the incorporation of PP at volume fractions of 0.2% decreased the compressive strength by 9.2% and 11.32% compared with plain concrete at age of 28 and 90 days, respectively. For the concrete reinforced with BF, the compressive strength first increases and then decreases with increasing fibre content, and it reaches the maximum which increases by 2.6% in comparison to the control mixture when BF content is 0.1%. A similar tendency in compressive strength of concrete containing sole BF was also reported by Borhan [43] and Wang and Zhang [44], and the maximum compressive strength was achieved when the BF volume content was 0.3% and 0.1%, respectively.

In general, the addition of fibres introduces defects, including bubbles and loose matrix in the concrete. More specifically, the defects are the poor fibre-interfacial transition zone (fibre-ITZ) with high porosity. These lead to a decrease in the compactness of concrete, which has a negative effect on compressive strength, especially at a higher fibre content due to uneven dispersion. However, evenly distributed fibres will form a dimensional network structure that can not only transport or dissipate stress well, but also effectively overcome the relative slip between the particles, and connect the damaged parts into a whole. Moreover, the bridge-action of fibre delays the formation and propagation of microcracks in matrix during cement hardening. The network structure and the bridge-action both show positive effects on the compressive strength. Once the positive effect overshadows the negative effect, the compressive strength will increase. On the contrary, the strength will decrease. BF has a more tangible positive effect than PP in compressive strength. This can be attributed to the fact that BF has a smaller diameter and there are more fibres per unit concrete volume at the same fibre length and volume content; a larger distribution density of BF may lead to a more powerful network structure.

It is worth mentioning that the maximum compressive strength is achieved in the hybrid mixture PP01BF01, which contains 0.1% PP and 0.1% BF. The compressive strength reaches 68.5 MPa which is 4.74% higher than that of no-fibre concrete, which seemingly does not comply the single fibre rules on compressive strength as analysed above. This phenomenon can be explained by the complementation of two type of fibres. The complementation of fibres can be reflected in size or elastic modulus. Under this proportion, the different sizes of two types of fibres may cause a relatively more complete network structure compared to one size. The PP with low elastic modulus and BF with high elastic modulus can enhance the bridge-action of fibres at different stage of cement hardening [28]. In addition, fibre may block capillary pores in concrete; therefore, PP and BF with different diameters may maximize the blocking-effect which leads to a densification of the concrete matrix.

### 3.3. Splitting Tensile Strength and Flexural Strength.

**Figure 5:**
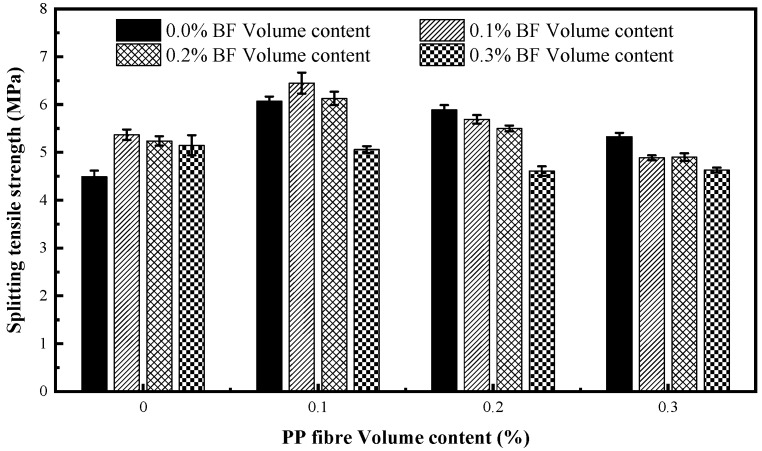
Splitting tensile strength of fibre-reinforced concrete.

Figure 5 gives the splitting tensile strength of the mixtures of different fibre volumes.

As expected, incorporating fibre significantly improved the splitting tensile strength of concrete, and the augmented percentage of the splitting tensile strength of fibrous mixtures is in the range of 2.67–43.65% in comparison to that of no-fibre concrete. The results are consistent with those analysed previously by other researchers [23,45,46,47]. This can be attributed to the bridge-action of fibres across cracks, which effectively delays and restrains the propagation of microcracks and macrocracks in the splitting tensile test. Nevertheless, no proportional relationship exists between the fibre content and the splitting tensile strength. Beyond the single PP or BF content of 0.1%, the flexural strength starts to decrease, but is still higher than the control mixtures. This can be attributed to the fact that the defects caused by fibres are intensified due to increasing fibre volume, and the positive effect of fibres is relatively reduced.

Compared with the control mixture, the splitting tensile strength of concrete reinforced with the single PP increases by 18.71–35.19%, and the splitting tensile strength of concrete reinforced with the single BF increases by 14.7–19.6%. It is easily observed that the addition of the single PP has a more significant influence on splitting tensile strength in comparison to BF, which is opposite to that observed for compressive strength. The same result in high performance concrete was reported by Smarzewski [9]; they indicated that short PP with a larger diameter can bridge the microcracks much more efficiently than BF. In this paper, the authors indicate that the bonding performance between PP and concrete matrix is better than that of BF, which is the main reason for a more obvious tensile strength enhancement in concrete reinforced with PP alone. In addition, a higher elongation at break of PP more than six times greater than that of BF is also conducive to this phenomenon.

The greatest enhancement in splitting tensile strength belongs to the mixture PP01BF01 with a content of 0.1% PP and 0.1% BF. This illustrates that the hybridization of PP and BF at a certain proportion can improve the splitting tensile strength of concrete in comparison to the individual fibres. This is mostly due to the complementary crack-arresting behaviour of the two fibres at different damage stages of the splitting tensile test. The BF with small diameter can delay the initiation and propagation of microcracks in the early stage of damage, and the PP with large diameter can control the formation of the macrocracks in the later stage. The complementation between PP and BF effectively inhibits the propagation of cracks in the concrete matrix, significantly improving the splitting tensile strength. However, there is no tangible increase of splitting tensile strength in other hybrid mixtures, which suggests the positive effect of hybridisation only occurs at a specific hybrid fibre proportion.

**Figure 6:**
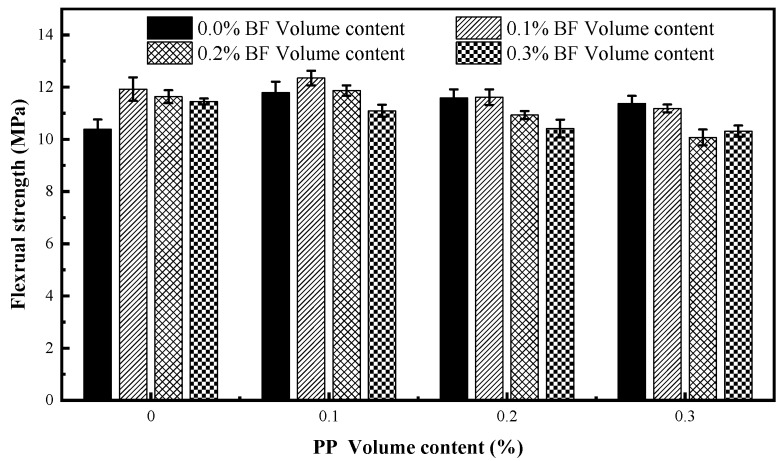
Flexural strength of fibre-reinforced concrete.

Figure 6 gives the results of flexural strength of all mixtures. The addition of fibres generally increases the flexural strength of concrete, and the strength ranges from 10.07 to 12.35 MPa. Compared to plain concrete, the flexural strength of concrete reinforced with the single PP increases by 9.54–13.85%, and the flexural strength of concrete reinforced with the single BF increases by 10.31–14.84%. Previous studies have demonstrated that the addition of the single PP and the single BF to concrete both considerably improved the flexural behavior of concrete [14,23,26]. The augmented percentage also shows there is no obvious difference of the efficiency in improving the flexural strength of concrete between the addition of the single PP and BF, which is different from the results for compressive and splitting tensile strength as mentioned above. Furthermore, the flexural strength is less sensitive to the addition of fibres than the splitting tensile strength, and shows lower increments at the same fibre volume, which indicates a more important role of fibres in tensile damage.

The maximum of flexural strength is similarly achieved in mixture PP01BF01. In this mixture, the compressive, splitting tensile and flexural strength improve by 4.74, 43.65 and 18.98% compared with those of concrete with no fibre, respectively. This indicates that the hybrid mixture containing 0.1% PP and 0.1% BF achieves the best mechanical performance.

A decrease of flexural strength occurred in hybrid mixtures PP03BF02 and PP03BF03, and the strength decreases by 2.99% and 0.67% in comparison to control mixture, which does not comply with the literature. This phenomenon can be attributed to the fact that the two types of fibres are intertwined with each other, and cause serious defects which considerably decrease the compactness of concrete. This suggests that the hybridization of fibres at high volume can have adverse influence on the mechanical properties of concrete.

### 3.4. The Synergy of PP and BF

As mentioned above, the hybridization of fibres at different fibre volume content has different influences on the mechanical properties of concrete. To further analyse this phenomenon, the synergy of PP and BF is proposed and divided into positive synergy effect and negative synergy effect. Hua and Zeng [48] reported the hybrid effect of steel fibre and PP, PP and carbon fibre in concrete, and an equation for calculating the hybrid coefficient of these fibres was proposed. Wang and Wu [49] defined the enhancement coefficient of hybrid fibre, and the enhancement coefficient of steel fibres with different geometric sizes were calculated. Based on previous research, the equations for calculating the synergy coefficients are optimized and defined as follows:

(1) $$\beta =\frac{f_{x}}{f_{x0}}$$

(2) $$\alpha_{f(x1)}=\frac{\beta_{PP-BF}{+\beta }_{min(PP,BF)}}{\beta_{PP}{+\beta }_{BF}}$$

(3) $$\alpha_{f(x2)}=\frac{\beta_{PP-BF}{+\beta }_{max(PP,BF)}}{\beta_{PP}{+\beta }_{BF}}$$

where, β is the strength effectiveness due to fibre, $f_{x}$ and $f_{x0}$ are the strength of fibre concrete and the strength of plain concrete; $\beta_{PP}$ and $\beta_{BF}$ are the strength effectiveness of the single PP and the single BF, respectively; $\beta_{min(PP,BF)}$ and $\beta_{max(PP,BF)}$ are the minimum and maximum of $\beta_{PP}$ and $\beta_{BF}$; $\beta_{PP-BF}$ is the strength effectiveness of hybrid fibre concrete; $\alpha_{f(x1)}$, $\alpha_{f(x2)}$ are the synergy coefficients; *c*, *sp* and *f* represents the compressive strength, splitting tensile strength and flexural strength, respectively. For instance, $\alpha_{f(c1)}$ and $\alpha_{f(c2)}$ are the synergy coefficients of the compressive strength; $\alpha_{f(sp1)}$ and $\alpha_{f(sp2)}$ are the synergy coefficients of the splitting tensile strength.

When $\alpha_{f(x1)}\geq 1$, the hybrid fibres exert a positive synergy effect; when $\alpha_{f(x1)}<1$, it needs to be judged according to $\alpha_{f(x2)}$. If $\alpha_{f(x2)}\geq 1$, it is still a positive synergy effect; if $\alpha_{f(x2)}\leq 1$, it is a negative synergy effect. The synergy coefficients of compressive strength, splitting tensile strength and flexural strength of hybrid fibre concrete are presented in Table 4.

The mixtures PP01BF01 and PP02BF01 both show positive synergistic effects on three types of strength, and PP01BF02 only shows a positive synergistic effect on splitting tensile strength and flexural strength. The positive synergy effect can be attributed to the complementation of PP and BF in terms of elastic modulus and elongation [9]. Additionally, two types of fibres with different diameter may block parts of capillary pores in concrete, which increases the compactness of concrete. Luo and Bi [50] indicated that the hybridization of PP and BF at a certain proportion can reduce the pore volume of concrete due to the fibre-blocking effect.

The mixtures PP01BF03, PP02BF03, PP03BF01, PP03BF02 and PP03BF03 all show negative synergistic effects on three types of strength. This indicates that the hybridization of PP and BF at total fibre volume above 0.3% exerts adverse effects on the mechanical properties. This phenomenon is mostly due to the intertwining and folding of the two types of fibres at higher volume, which considerably hinders the uniform dispersion of fibres in the concrete and causes serious defects. The serious defects overshadow the complementation of the two types of fibres; therefore, the hybridization shows a negative effect on three types of strength.

Nevertheless, the mixture PP02BF02 at a total fibre volume of 0.4% shows a positive synergistic effect on splitting tensile strength. This may be attributed the fact that fibres play a more important role in tensile damage in comparison to compressive and flexural damage as mentioned earlier, and the defects due to high fibre volume do not overshadow the complementation of the two fibres in the tensile test. Similarly, the defects easily overshadow the complementation in the compressive test. Therefore, a negative synergistic effect on the compressive strength of mixture PP01BF02 was observed, whereas the synergistic effect on the splitting tensile strength and flexural strength is positive.

In summary, the hybridization of PP and BF only in certain proportions can exert a positive synergistic effect. The data suggest that the total hybrid fibre volume content should be no more than 0.3%, and the volume content of BF should be no more than 0.1%. In this range, the hybrid fibres are more effective at improving the mechanical properties of concrete compared to the single fibres.

### 3.5. Chloride Resistance

**Figure 7:**
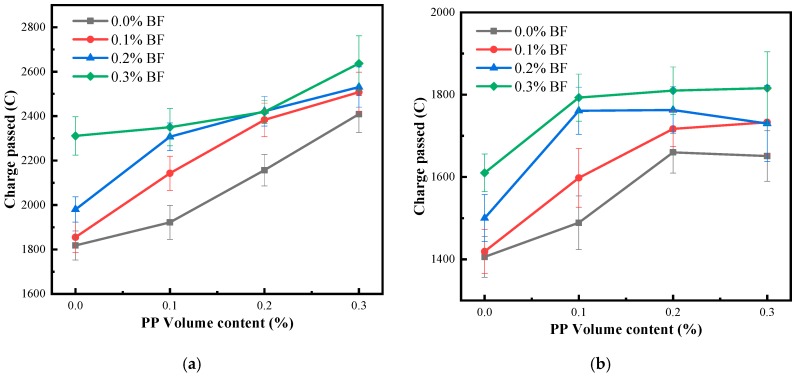
The charge passed by concrete: (**a**) at the age of 28 days; (**b**) at the age of 56 days.

The charge passed by all mixtures at 28 and 56 days is given in Figure 7.

The addition of fibres increased the charge of mixtures, and the addition of the single PP always has a more obvious charge increase than the addition of BF. The charge of concrete is in the range of 1818–2638 Coulomb at 28 d and 1406–1816 Coulomb at 56 d and the minimum charge always belongs to the mixture without fibre. At the age of 28 days, the incorporation of the single PP at volume fractions of 0.1–0.3% increases the charge of concrete by 5.72–32.51%, and the incorporation of the single BF at volume fractions of 0.1–0.3% increases the charge of concrete by 2.04–27.12%. At the age of 56 days, the corresponding increase percentage is 0.92–14.15% and 5.9–17.43%, respectively.

Susanto et al. [51] indicated that the addition of fibres can lead to an increase in the chloride migration coefficient of concrete. Sadrmomtazi et al. [35] reported that the presence of BF in cementitious materials can cause increasing water absorption and decreasing electrical resistivity. Gong and Guo [52] and Toutanji et al. [34] indicated that the inclusion of PP decreased the resistance of concrete to chloride penetration. The increase in charge can be attributed to the defects caused by fibres. The defects include polyporous fibre-ITZ, gaps after fibre debonding, and voids which are difficult to eliminate due to the decrease of the workability of the concrete. Moreover, the defects caused by fibres will be intensified at a high fibre volume due to entangling and folding of fibres. All of these can increase the porosity of concrete, which increases chloride diffusion. Besides, although PP has a better bonding performance than BF, the workability of concrete fabricated with the single PP is lower than that of the single BF at the same fibre content, as shown in Table 3. This leads to a greater charge increase in concrete reinforced with PP alone.

It is important to note that the hybridization of two types of fibres has no significant effect on the chloride resistance of concrete; even hybridization at a high fibre volume will intensify the charge increase of concrete. This also indicates that the hybridization of PP and BF may have limited influence on the pore structure of concrete.

Furthermore, the addition of fibres increases the charge of concrete by 2.04–45.05% in comparison to the mixture without fibre at 28 days, and the corresponding increase is in the range of 0.92–29.16% at 56 days. This indicates the efficiency of fibres in increasing the charge of concrete is decreased at a later age. In other words, the increase of the curing age mitigates the adverse effect of fibres on chloride resistance. The reasons can be summarized as follows: first, the hydration reaction of cement can reduce calcium hydroxide (CH) with preferential orientation around the fibres, which improves the loose matrix around the fibres; second, the formation of large amounts of calcium silicate hydrate (C-S-H) gels can improve the fibre-ITZ, and significantly enhance the fibre bonding performance. These all mitigate the defects caused by fibres.

### 3.6. Morphology

**Figure 8:**
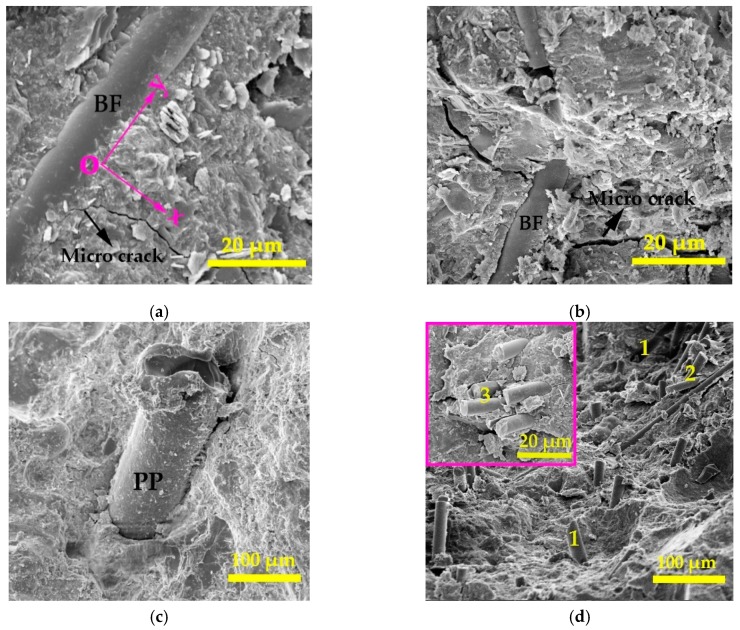
Morphology of PP and BF in the concrete matrix. (**a**) PP0BF01; (**b**) PP0BF03; (**c**) PP01BF0; (**d**) PP03BF03.

Some representative images of composites with different fibres are shown in Figure 8.

In Figure 8a,b, the cracks are clear, and the cracks may be formed by the shrinkage of the concrete matrix, or by sampling before SEM. In addition, the wall of BF is smooth and is not attached to obvious hydration products. The presence of fibre in Figure 8a changes the propagation direction of microcracks (the x axis to the y axis), then controls the continued opening of cracks. The fibre across the penetrating crack in Figure 8b can arrest the width of the advancing crack which reflects the bridge-action. These factors result in improvement of the mechanical strength of concrete.

Compared with Figure 8a,b, Figure 8c has a lower magnification but shows a larger fibre size, which indicates a higher surface area of PP than BF. Moreover, the surface of PP is much rougher and the hydration products that appear as small rice shapes around the fibre are clear. The rough wall of PP increases the friction between the fibre and the matrix, and indicates a higher fibre bonding performance of PP than BF. The fibre reinforcement mechanism in tensile damage is usually supplemented by the energy consumption of pulling-out but not fracture of fibres. The pulling-out of fibres due to excessive shear friction mainly depends on the bonding between the fibre and matrix. Thereby, the addition of PP is more effective for improving the tensile strength of concrete in comparison to the addition of BF.

In Figure 8d, at a highly hybrid fibre volume, clumping, debonding and kinking are observable. This indicates the hybridization of fibres at a high volume causes serious defects, which is negative to the mechanical strength and chloride resistance properties of concrete. The numbers in images indicate: (1) debonding, (2) clumping, (3) kinking.

### 3.7. Pore Structure

**Figure 9:**
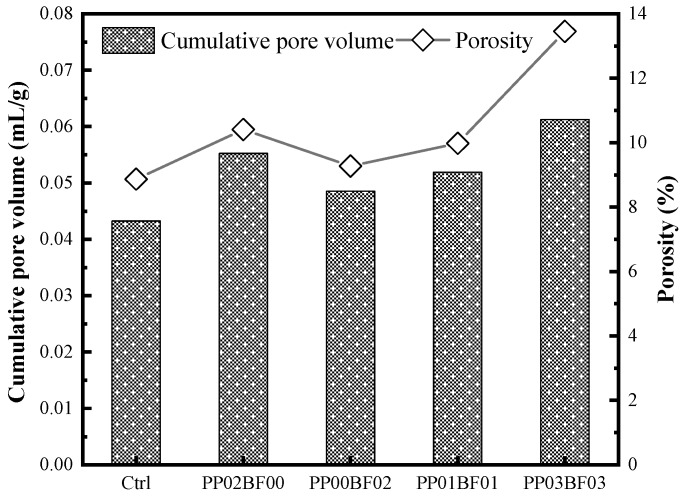
The cumulative pore volume and porosity.

To analyse the influence of PP and BF on the pore structure, singly or in hybridization, the mixtures Ctrl, PP02BF00, PP00BF02, PP01BF01 and PP03BF03, cured for 28 days, were chosen as samples. The cumulative pore volume and porosity of theses samples are shown in Figure 9. The results show that the addition of fibres increases the cumulative pore volume and porosity of concrete, and a higher value is obtained in higher fibre volume. Zhang et al. [53] reported that incorporating BF not only increases the quantity of harmful pores, but also introduces some internal microcracks, weakening the interfaces. Deng et al. [54] indicated that the addition of PP increases the porosity of cement mortar, and the porosity of mortar when the PP volume fraction is 0.3% increases by 17.74% in comparison to that with no fibre. Other organic fibres, such as polyvinyl alcohol fibres and polyolefin fibre, were investigated in previous studies, which indicated a higher porosity of concrete reinforced with these fibres compared to concrete with no fibre [39,51]. The increase in pore volume and porosity can be attributed to polyporous fibre-ITZ and gaps after deboning of fibre. Guo et al. [55] indicated that the defects introduced by fibres are essentially a loose matrix formed around the fibre. Additionally, the voids and bubbles in concrete are challenging to eliminate by vibration due to the decrease in workability, which intensifies this adverse effect.

The hybridization of PP and BF in improving the pore structure of concrete was not observed, which indicates that the mechanical strength enhancement of the hybrid mixtures is mainly due to the complementation of PP and BF in terms of diameter, elastic modulus and elongation. The increase in charge of hybrid mixture also indicates that the hybridization of fibres has no significant influence on the pore structure of concrete.

**Figure 10:**
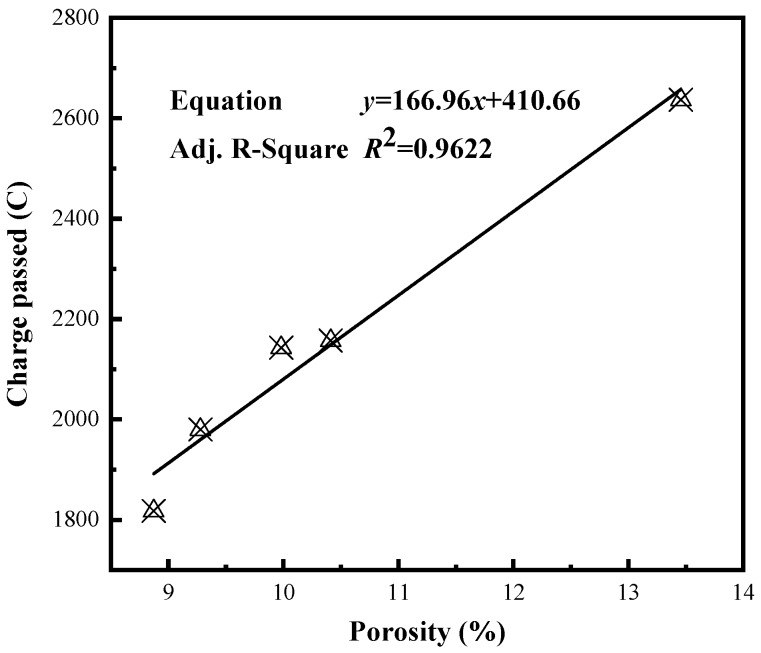
Relationship between the porosity and charge of concrete.

Figure 10 gives the relationship between the porosity and charge of concrete. Increased porosity is associated with increased charge. The determination coefficient of this relation is 0.9622, indicating a strong correlation between the two factors.

**Figure 11:**
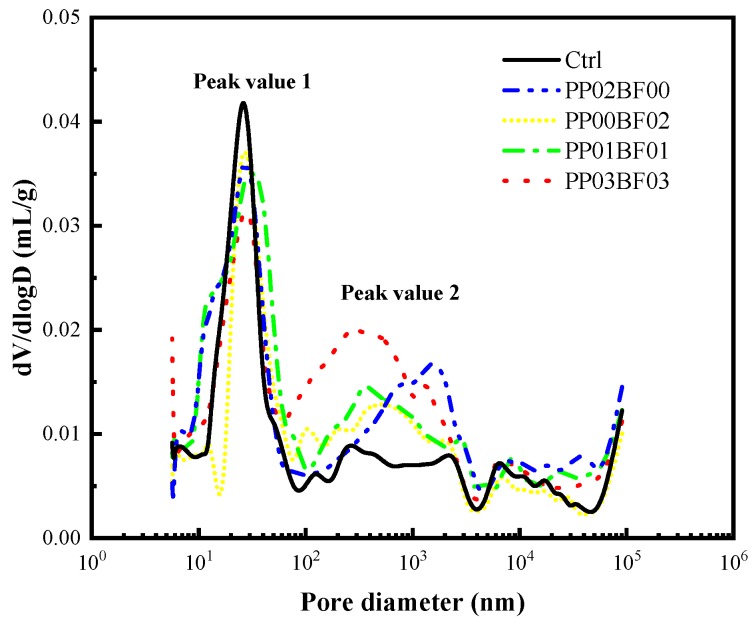
Pore size distribution curve.

The variations in the pore size distribution of the samples are presented in Figure 11. It can be seen that only one peak is observed in the control, while another peak appeared when fibre was added. With the increase of total fibre volume, peak value 1 decreased, and peak value 2 increased. This indicates that the addition of fibres changes small-size pores into large-size pores. The reason may be that the addition of fibres transforms the original pore structure, and pores may interconnect along the length of the fibres and become larger. The interconnection of pores forms a perforated and continuous network structure that is smoother for diffusion, which results in decreased chloride resistance of concrete. Alternatively, the fibres may block parts of pores and then reduce the small-size pores, but the defects due to the fibres considerably increase the large-size pores.

Furthermore, peak 2 is moving to the left of the pore diameter axis upon hybridization of two types of fibres, as shown in Figure 11. This indicates that the hybridization of PP and BF can improve the pore size distribution in the concrete, compared with single fibres at the same total volume content. This may be attributed to the difference of diameter between the two types of fibres. However, the improvement in the pore size distribution has no significant effect on the macro-performance of concrete.

## 4. Conclusions

The workability, mechanical performance, chloride resistance, interface and pore structure of concrete containing the PP or BF and their hybridization were investigated. Based on the test results, the following conclusions can be drawn:

1. The hybridization of PP and BF has no significant influence on the workability of concrete compared to the single fibres at the same content.
2. The combination of PP and BF can improve the compressive, splitting tensile and flexural strength of concrete in comparison to the individual fibres. However, the hybridization is not always conductive, and it has positive and negative synergistic effects at different volume proportions. It is suggested that the total hybrid fibre volume content should be no more than 0.3%, and the volume content of the single BF should be no more than 0.1%. In this paper, the optimum combination is 0.1% PP and 0.1% BF by volume of concrete, and the compressive, splitting tensile and flexural strength were improved by 4.74, 43.65 and 18.98% compared with those of no-fibre concrete, respectively.
3. Incorporating fibres reduced the chloride resistance of concrete, and a more obvious reduction was observed when adding PP in comparison to adding BF. The hybridization of the two fibres does not enhance this phenomenon; however, the reduction was intensified at high hybrid fibre volume. Increasing the curing age can mitigate the adverse effect on the chloride resistance of concrete caused by fibres.
4. The addition of PP and BF, singly or in hybridization, increases the cumulative pore volume and porosity of concrete. The fibres transform the original pore structure and changes small-size pores into large-size pores. Moreover, the hybridization of fibres can improve the pore size distribution in the concrete compared with the single fibres at the same volume.

The findings of this paper expand the knowledge of FRC and provide a reference for the application of concrete reinforced with polypropylene and basalt fibres in civil engineering. However, further research is needed to explore the influence of hybridization of polypropylene and basalt fibres on other properties of concrete, particularly on durability and dynamic performances.

## Figures and Tables

**Table 1:** Main chemical composition and physical properties of cement.

The Chemical Composition (%)								The Physical Properties	
SiO_2_	Al_2_O_3_	Fe_2_O_3_	Na_2_O	MgO	K_2_O	CaO	MnO	Specific Gravity (Kg/m^3^)	Specific Surface (m^2^/Kg)
20.94	2.84	4.64	0.48	1.65	0.26	69.03	0.16	3150	350

**Table 2:** Physical and mechanical properties of BF and PP.

Type	Length (mm)	Diameter (μm)	Density (g/cm^3^)	Tensile Strength (MPa)	Elastic Modulus (GPa)	Elongation (%)
PP	12	18–45	0.91	310–540	3.72	20
BF	12	16	2.65	2630	88.9	2.99

**Table 3:** Mixture proportions.

Mix ID	Cement	Water	Fine Aggregate	Coarse Aggregate	SP	PP	BF	Slump
	(Kg/m^3^)					(%)		(mm)
Ctrl	500	160	1044	696	4	0.0	0.0	130
PP01BF0	500	160	1044	696	4	0.1	0.0	100
PP02BF0	500	160	1044	696	4	0.2	0.0	85
PP03BF0	500	160	1044	696	4	0.3	0.0	65
PP0BF01	500	160	1044	696	4	0.0	0.1	120
PP0BF02	500	160	1044	696	4	0.0	0.2	105
PP0BF03	500	160	1044	696	4	0.0	0.3	80
PP01BF01	500	160	1044	696	4	0.1	0.1	100
PP01BF02	500	160	1044	696	4	0.1	0.2	80
PP01BF03	500	160	1044	696	4	0.1	0.3	65
PP02BF01	500	160	1044	696	4	0.2	0.1	75
PP02BF02	500	160	1044	696	4	0.2	0.2	65
PP02BF03	500	160	1044	696	4	0.2	0.3	55
PP03BF01	500	160	1044	696	4	0.3	0.1	60
PP03BF02	500	160	1044	696	4	0.3	0.2	55
PP03BF03	500	160	1044	696	4	0.3	0.3	50

Note: the percentage of fibres is by volume fraction of concrete; 0.1% polypropylene fibre and basalt fibre in Table are 0.92 kg/m^3^ and 2.65 kg/m^3^, respectively.

**Table 4:** The synergy coefficient.

Mix ID	$\alpha_{f(c1)}$	$\alpha_{f(c2)}$	$\alpha_{f(\mathrm{sp}1)}$	$\alpha_{f(\mathrm{sp}2)}$	$\alpha_{f(f1)}$	$\alpha_{f(f2)}$
PP01BF01	1.011	–	1.033	–	1.018	–
PP01BF02	0.970	0.998	1.005	–	1.003	–
PP01BF03	0.976	0.988	0.910	0.992	0.970	0.985
PP02BF01	0.955	1.048	0.982	1.028	0.987	1.001
PP02BF02	0.924	0.983	0.965	1.023	0.970	0.972
PP02BF03	0.959	0.978	0.884	0.951	0.949	0.955
PP03BF01	0.884	0.999	0.955	0.959	0.968	0.992
PP03BF02	0.880	0.963	0.968	0.959	0.932	0.943
PP03BF03	0.893	0.937	0.950	0.933	0.950	0.954
